# Tumor-associated macrophage expression in colorectal adenomas and carcinomas: relationship to *Helicobacter pylori* infection

**DOI:** 10.3389/fonc.2025.1649619

**Published:** 2025-11-28

**Authors:** Wenming Wang, Yueyong Zhu, Yunchao Zhu, Jin Wang

**Affiliations:** 1 Liver Disease Diagnosis and Treatment Center, The First Affiliated Hospital of Fujian Medical University, Fuzhou, Fujian, China; 2 Department of Gastroenterology and Pathology, Affiliated Hospital of West Anhui Health Vocational College, Lu’an, Anhui, China

**Keywords:** CD163, CD86, colorectal adenomas, colorectal cancer, *Helicobacter pylori*

## Abstract

**Objective:**

This study investigated the association between *Helicobacter pylori (H. pylori)* infection and the expression of CD163^+^ and CD86^+^ tumor-associated macrophages (TAMs) in colorectal adenoma (CRA) and colorectal cancer (CRC) tissues.

**Methods:**

Immunohistochemistry (IHC) was used to evaluate the expression of CD163^+^ and CD86^+^ TAMs isolated from colorectal tissues, Multiplex immunofluorescence (mIF) co-staining was employed to identify CD68^+^CD163^+^ and CD68^+^CD86^+^ TAMs, and the ^14^C-urea breath test (UBT) was used to detect *H.pylori* infection.

**Results:**

The progression of colorectal lesions was significantly associated with increased expression of CD163^+^ and CD86^+^ TAMs, as well as *H.pylori* infection (all *P <* 0.05). The expression of CD163^+^ and CD86^+^ TAMs were positively correlated with each other and with the severity of colorectal lesions (all *P <* 0.001). Patients with *H.pylori* infection exhibited significantly higher expression of both TAM subsets compared with non-infected individuals (all *P <* 0.05). Multiple linear regression analysis showed that in colorectal adenomas measuring ≥ 1 cm, expression of CD163^+^ and CD86^+^ TAM was significantly greater than in adenomas <1 cm (*P <* 0.05), Expression of CD163^+^ TAM was notably higher in obese patients with CRC. Multiplex immunofluorescence (mIF) quantification revealed significantly increased densities of both CD68^+^CD86^+^ and CD68^+^CD163^+^ TAMs, and a higher CD68^+^CD163^+^/CD68^+^CD86^+^ ratio in colorectal cancer (CRC) (all *P <* 0.001).

**Conclusions:**

The expression of CD68^+^CD163^+^ and CD68^+^CD86^+^ TAMs change dynamically with the progression of colorectal lesions. These changes are influenced by *H.pylori* infection, adenoma size, tumor differentiation, and patient metabolic status.

## 1 Introduction

Colorectal cancer (CRC) is a common malignant tumor of the digestive system. According to global cancer statistics in 2020, the incidence and mortality of CRC both rank third among all malignant tumors (1). The pathogenesis of CRC is associated with multiple factors, including genetic predisposition, environmental influences, lifestyle, and the gut microbiota. Notably, dysbiosis of the gut microbiota or infection with certain bacteria, such as *Helicobacterpylori* (*H. pylori*), can increase the risk of colorectal tumors (2). In addition, Gut microbiota linked to obesity is implicated in the pathogenesis of colorectal cancer (3). This is an important issue that warrants considerable attention.

Colorectal adenomas (CRA) mainly include two major categories: conventional adenomas(CAs) and sessile serrated adenomas (SSAs). CAs are further subdivided into tubular adenomas, villous adenomas, and tubulovillous (mixed) adenomas, each with distinct histological characteristics and malignant potential. SSAs are characterized by a unique serrated glandular architecture and a higher rate of malignant transformation, distinguishing them from CAs. Both types of adenomas carry a risk of malignant progression (4) and are recognized as precancerous lesions. Timely detection and intervention can reduce the risk of CRC development. Although colorectal cancer (CRC) treatment employs a multidisciplinary, multimodal integrated strategy (5), a proportion of patients present with advanced disease at diagnosis, having missed the optimal window for radical treatment. Consequently, their overall prognosis is significantly worse than that of early-stage patients (Stage I patients exhibit a > 90% 5-year survival rate) (6). Thus, there is an urgent need to identify novel tumor biomarkers that can predict tumor progression and serve as potential therapeutic targets.

The tumor microenvironment (TME) is a complex and dynamic ecosystem with high heterogeneity, Macrophages within the TME, known as tumor-associated macrophages (TAMs), are important immune effector cells primarily derived from peripheral blood monocytes and tissue-resident macrophages. TAMs consist mainly of two subsets, M1 and M2 (7). The M1 phenotype generally inhibits tumor growth, whereas the M2 phenotype promotes tumor growth. The balance between both subsets determines the nature of the immune response in the TME.

Cell surface markers of TAMs primarily include CD68 (pan-macrophage), CD86 (M1-like macrophage markers), and CD163 (M2-like macrophage markers) (8, 9). In the TME of CRC, the polarization state of TAMs is associated with cancer-specific survival, and M1-like/M2-like TAMs phenotypes exhibit distinct prognostic implications (10). Targeting TAMs as a therapeutic strategy has already become an effective approach to inhibit tumor progression (11). Nonetheless, the mechanisms by which TAMs evolve within the TME during the malignant progression of CRA are still not completely understood.

*H.pylori* infection can promote tumor development by influencing TAMs polarization in the TME. For example, sit. et al. (12) found that *H.pylori* phospholipase A regulates macrophage autophagy and apoptosis via the TNFR1-mediated p38 signaling pathway. Lu et al. (13) reported that interactions between reactive oxygen species and hypoxia-inducible factor-1α modulate *H.pylori*–induced macrophage polarization through the Akt/mTOR pathway. In addition, *H.pylori* infection can upregulate the expression of indoleamine 2,3-dioxygenase in macrophages, thereby inducing M2-like polarization (14). M2-like TAMs secrete various growth factors, enabling tumor cells to evade immune surveillance and elimination. Therefore, further investigation of the impact of *H.pylori* infection on TAMs polarization in CRA and CRC tissues is warranted.

In this study, we analyzed the expression of CD163^+^ and CD86^+^ TAMs in CRA and CRC tissues and examined their correlation with *H.pylori* infection, with the aim of providingreferences for clinical research.

## 2 Materials and methods

### 2.1 Study population

This study included patients diagnosed with CRA or CRC at the Affiliated Hospital of West Anhui Health Vocational College between January and December 2023. A total of 109 patients were enrolled, and clinical data and colorectal tissue specimens were collected. Among these, 61 patients had CRA, including 36 with CAs and 25 with SSAs. Among the 36 patients with CAs, 18 were tubular adenomas and 18 were tubulovillous adenomas. The study also included 29 patients with CRC and 19 individuals with normal colorectal mucosa who served as control. The normal control group comprised asymptomatic volunteers. Inclusion required: (i) endoscopically normal colonic mucosa with histopathological confirmation, (ii) no antibiotics 3 months/PPIs 1 month pre-enrollment, (iii) verified *H. pylori*-negative status. This study employed the urea breath test (UBT) as the sole diagnostic criterion for *H.pylori* infection. While UBT offers standardized operational advantages, the lack of orthogonal verification methods (e.g., histopathology, stool antigen testing, or serology) may lead to misclassification of infection status, thereby introducing unquantified classification bias.

#### 2.1.1 Inclusion criteria

The following inclusion criteria were applied to patients for this study: (i) tissue specimens with a definitive pathological diagnosis of either a CAs, a SSA, or CRC. The diagnostic criteria for SSA were crypt distortion and dilation with basal expansion forming a serrated architecture that extends along the muscularis mucosae, resulting in an inverted T-shaped or L-shaped configuration (15); (ii) availability of complete clinical and pathological data were available for each patient, including sex, age, tumor stage, tumor differentiation, lymph node metastasis status, and tumor location; (iii) *H.pylori* testing was performed in accordance with consensus guidelines. All subjects confirmed no history of eradication therapy and documented discontinuation of antibiotics/bismuth agents (≥ 4 weeks) as well as PPIs/acid suppressants (≥ 14 days) prior to sampling, A ^14^C-urea breath test (UBT) value ≥ 100 decays per minute (dpm) is defined as the positive diagnostic threshold for active *H.pylori* infection; and (iv) informed consent provided for the collection and use of their clinical data.

#### 2.1.2 Exclusion criteria

The following patients were excluded: (i) patients with severe cardiovascular, respiratory, or hematological diseases; (ii) those who had received any non-surgical treatments (such as radiotherapy, chemotherapy, targeted therapy, or immunotherapy); and (iii) patients with incomplete clinical or pathological data. The study was approved by the ethics committee of the Affiliated Hospital of West Anhui Health Vocational College (Approval No. LAEY-2022-017).

### 2.2 Main reagents and equipment

The following reagents and equipment were used in this study: mouse monoclonal anti-CD163 antibody (10D6, ma5-11458, Invitrogen, Waltham, MA02451, USA); rabbit monoclonal anti-CD86 antibody (EP1158-37, ab269587, Abcam, Cambridge, UK); rabbit Polyclonal anti-CD68 antibody (GB113150, Servicebio, Wuhan, China); rabbit Polyclonal anti-CD163 antibody (GB113152, Servicebio, Wuhan, China); rabbit Polyclonal anti-CD86 antibody (GB115630, Servicebio, Wuhan, China); secondary antibodies and DAB chromogenic kits (Biomiky, Biosharp, and Servicebio, respectively). ^14^C-urea breath test detector (Haidwei HUBT-20A2); and Haidwei disposable gas collection cards. Positive control tissues: (i) CD68^+^ and CD163^+^ macrophages: Human spleen (red pulp region); (ii) CD86^+^ macrophages: Human tonsil (germinal center). For validation, three randomly selected cases per cohort were analyzed by: (i) IHC quantification via H-score (range 0-300); (ii) Dual-color mIF confirming cellular co-expression in lesional tissue.

### 2.3 Immunohistochemistry

Specimens were fixed in formalin, embedded in paraffin, and sectioned into 3 μm-thick slices, Heat mediated antigen retrieval with Tris-EDTA buffer (pH 9.0) at 98 °8 for 20 minutes, Primary antibodies against CD163 (1:50) and CD86 (1:100) were applied after dilution, and slides were incubated at 4 °C, Secondary antibody incubation was conducted at room temperature for 15 min, followed by DAB staining for 4 minutes, Hematoxylin counterstaining and gradient alcohol dehydration were performed, and sections were mounted using neutral resin. Yellow-brown or tan granular staining was interpreted as positive expression. Two independent researchers evaluated the samples, For each slide, five randomly selected non-overlapping fields were analyzed at 200× magnification, The integrated optical density (IOD) of positive staining was quantified using ImageJ software, The mean IOD was calculated and used as the final result for each sample (16, 17).

### 2.4 Multiplex immunofluorescence

Twenty formalin-fixed, paraffin-embedded (FFPE) colorectal tissue samples were analyzed across three groups: normal mucosa (n=4), The CRA cohort included 10 cases: 5 CAs (3 tubular, 2 tubulovillous) and 5 SSA, and CRC (n=6). For each specimen, two consecutive sections were prepared and subjected to immunofluorescence staining: one for dual detection ofCD68^+^CD163^+^ TAMs, and the other for CD68^+^CD86^+^ TAMs. Double-positive macrophage density was quantified by dual-channel co-localization analysis (Pearson’s coefficient ≥ 0.6) in ImageJ. For each specimen, three anatomical zones were defined: Central zone (tumor core ± 1 mm), Marginal zone (invasive front ± 500 μm),Adjacent mucosal zone (≥ 2 mm from the margin). Cell counting primarily focused on stromal and combined zones, using hexagonal grid systematic sampling with 9 non-overlapping fields per zone (200 x magnification). Field distribution satisfied spatial uniformity testing (K-S p > 0.05). Data were normalized to cells per square millimeter (cells/mm²). Observer Agreement: Two pathologists independently counted 100% of specimens in a blinded manner. Intraclass Correlation Coefficient (ICC) = 0.92 (95% CI 0.88-0.95), Bland-Altman analysis: Mean bias + 1.5 cells/mm² (95% LoA-6.2 to 9.3), Results exceeded the prespecified reliability threshold (ICC ≥ 0.85).

### 2.5 Statistical analysis

All statistical analyses were conducted using SPSS v.27.0 software. Continuous variables conformed to normal distribution (Shapiro-Wilk p > 0.05), presented as [M ± SD], One-way ANOVA was employed to analyze intergroup differences (effect size ηp2), If results were significant (p < 0.05), Tukey HSD method was applied for pairwise comparisons (controlling FWER at α = 0.05). Continuous variables failing normality tests (Shapiro-Wilk p < 0.05) were summarized by medians and IQRs, defined as [Q1, Q3], Group differences were assessed using the Kruskal-Wallis H-test for three or more groups, Upon significance (p<0.05), pairwise localization employed Dunn-Bonferroni-adjusted (DB-adjusted) *post hoc* procedures, Comparisons between exactly two independent groups utilized the Mann-Whitney U test (MWU test). Categorical variables are presented as n (%), Intergroup comparisons were performed using Pearson’s χ²-test or Fisher’s exact test based on expected frequencies. Correlations between variables were assessed using Spearman’s rank correlation coefficient. A *P*-value < 0.05 was considered statistically significant. Multiple linear regression analysis was performed to identify independent factors associated with CD163^+^ and CD86^+^ TAMs expression. The regression model was specified as follows:


$$\text{Y}={\text{β}}_{0}+{\text{β}}_{1}{\text{X}}_{1}+{\text{β}}_{2}{\text{X}}_{2}+\ldots +{\text{β}}_{\text{k}}{\text{ X}}_{k}+\text{ϵ}$$

where Y represents the expression level of CD163^+^ or CD86^+^ TAMs (as integrated optical density, IOD), β_0_ is the intercept, β_1_ to β_1_ are regression coefficients for the independent variables X_1_ to X_1_, and ϵ is the error term. The model assumptions were checked using residual plots and the Shapiro-Wilk test. No severe multicollinearity was detected (all variance inflation factors < 5).

Prior to constructing the multivariate linear regression model, we first performed univariate analyses to screen clinical variables associated with TAMs expression. Covariates ultimately included in the model comprised variables with P-values < 0.05 in the univariate analyses, as well as important potential confounders identified based on clinical knowledge and expert judgment. Accordingly, factors such as adenoma size, adenoma number, obesity, tumor differentiation grade, lymph node metastasis, TNM stage, and *H. pylori* infection were collectively included in the final model. This strategy ensured that the model incorporated both statistical associations from the data and biological plausibility, thereby enabling a more accurate estimation of the independent associations between these factors and TAMs expression.

Given the relatively small sample size in the CRC group, the precision and statistical power of the model may be limited. This issue was addressed by using bidirectional stepwise regression to optimize variable selection and minimize overfitting, with results presented with 95% confidence intervals to reflect uncertainty.

## 3 Results

### 3.1 Expression of CD163^+^ TAMs, CD86^+^ TAMs, and *H. pylori* infection

**Figure 1:**
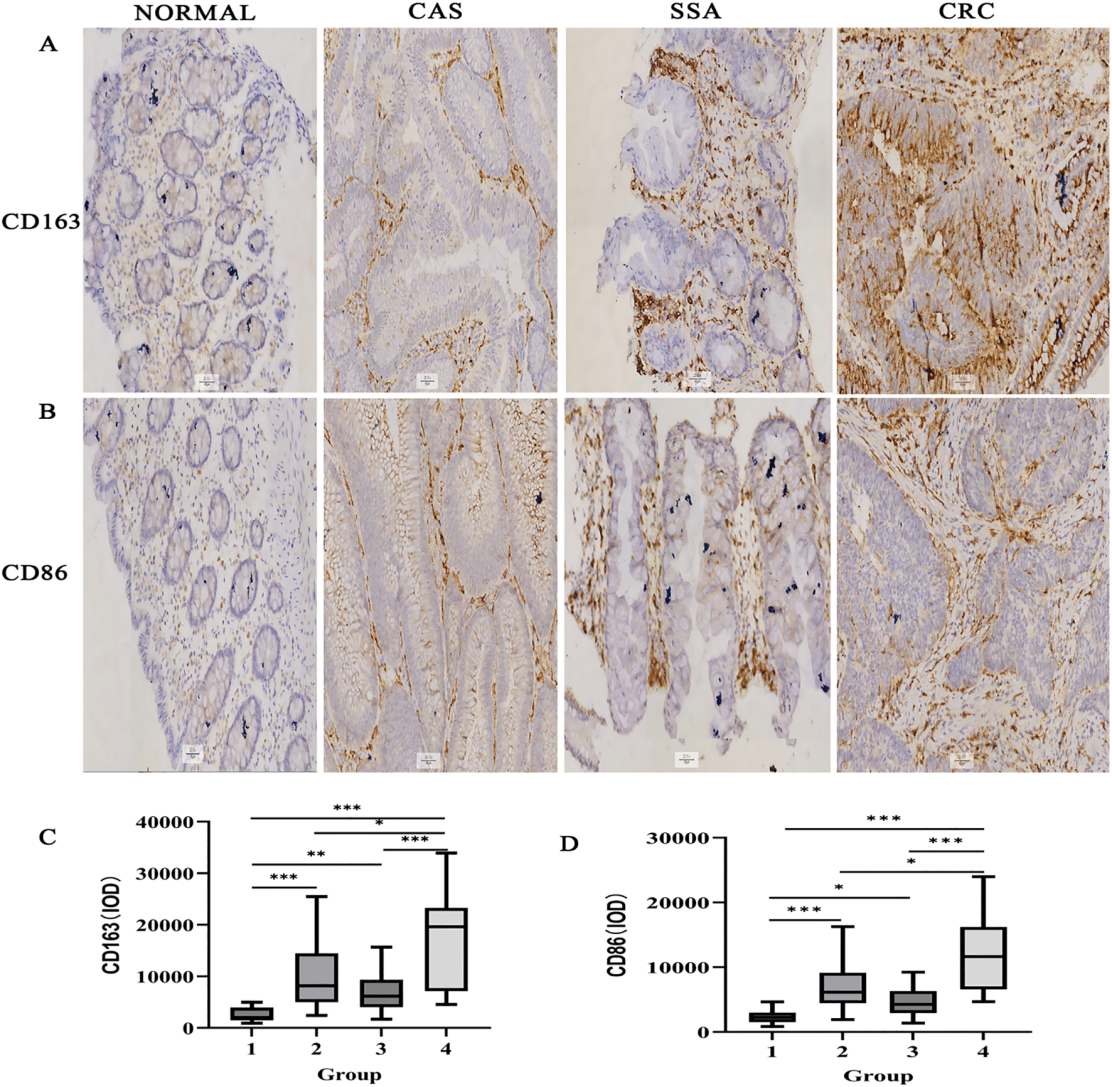
Immunohistochemical detection of CD163^+^ and CD86^+^ TAMs in colorectal tissues (×200). **(A)** CD163 staining in various tissues. **(B)** CD86 staining. **(C)** IOD for CD163. **(D)** IOD for CD86. Data expressed as M (Q1, Q3) (n = 109). *P < 0.05, **P < 0.01, ***P < 0.001 (DB-adjusted). Groups: 1, Normal; 2, CAS; 3, SSA; 4, CRC. M: Median; Q_1_: 1st Quartile; Q_3_: 3rd Quartile.

CD163^+^ and CD86^+^ TAMs were primarily located in the stromal matrix and appeared as brownish-yellow or tan granules. Their expression increased progressively with colorectal malignancy, from normal tissue to CRC, indicating a potential immunological role in tumor progression (**Figures 1A, B**).

Statistical analysis revealed significant differences in the expression of CD163^+^ and CD86^+^ TAMs among the normal, CAs, SSA, and CRC groups (all *P <* 0.05) (**Figures 1C, D**). DB-adjusted for pairwise comparison showed significantly higher expression in CRC than in tissues from CAs and SSA groups, and higher levels in CAs and SSA than tissues in the normal group (all *P <* 0.05). No significant difference was found between the CAs and SSA groups (*P* > 0.05). The overall trend in tissue expression was as follows: normal < CRA < CRC (*P <* 0.05) (**Table 1**).

**Table 1:** Expression of CD163^+^/CD86^+^ TAMs and *H. pylori* infection rates across groups.

		CRA(n=61)				
Variables	Normal (n=19)	CAs (n=36)	SSA (n=25)	CRC (n=29)	Statistic	P
CD163,M (Q_1_,Q_3_)	2066.45 (1492.80,3769.28)	8195.20 (5009.03,14448.74)^#^	6134.79 (4148.07,9337.56)^#^	19614.60 (7182.47,22899.10)^#△^	χ²=51.913^⋆^	<0.001
CD86,M (Q_1_,Q_3_)	2227.46 (1590.13,2721.17)	6139.86 (4491.34,8963.26)^#^	4261.13 (3055.10,5898.95)^#^	11644.53 (6618.20,15188.14)^#△^	χ²=61.431^⋆^	<0.001
*H.pylori*,n(%)					χ²=33.755	<0.001
Uninfected	18 (94.74)	18 (50)^#^	14 (56.00)^#^	3 (10.34)^#△^		
Infected	1 (5.26)	18 (50)^#^	11 (44.00)^#^	26 (89.66)^#△^		

⋆: Kruskal-Wallis test; M: Median; Q_1_: 1st Quartile; Q_3_: 3rd Quartile. DB-adjusted/Pearson’s χ² test: #*P <* 0.05 vs. normal, △*P <* 0.05 vs. CRA.

*H.pylori* infection rates also differed significantly among groups (*P <* 0.05). Bonferroni-corrected partitioned chi-square method for Pairwise comparisons indicated a higher rate in CRC compared with CAs and SSA, and higher in CAs and SSA compared with the normal group (all *P <* 0.05). There was no significant difference between CAs and SSA (*P* > 0.05). The overall trend in the infection rate was CRC > CRA > Normal (*P <* 0.05) (**Table 1**).

### 3.2 Association between CD163^+^/CD86^+^ TAMs, *H. pylori* infection, and colorectal malignancy

**Figure 2:**
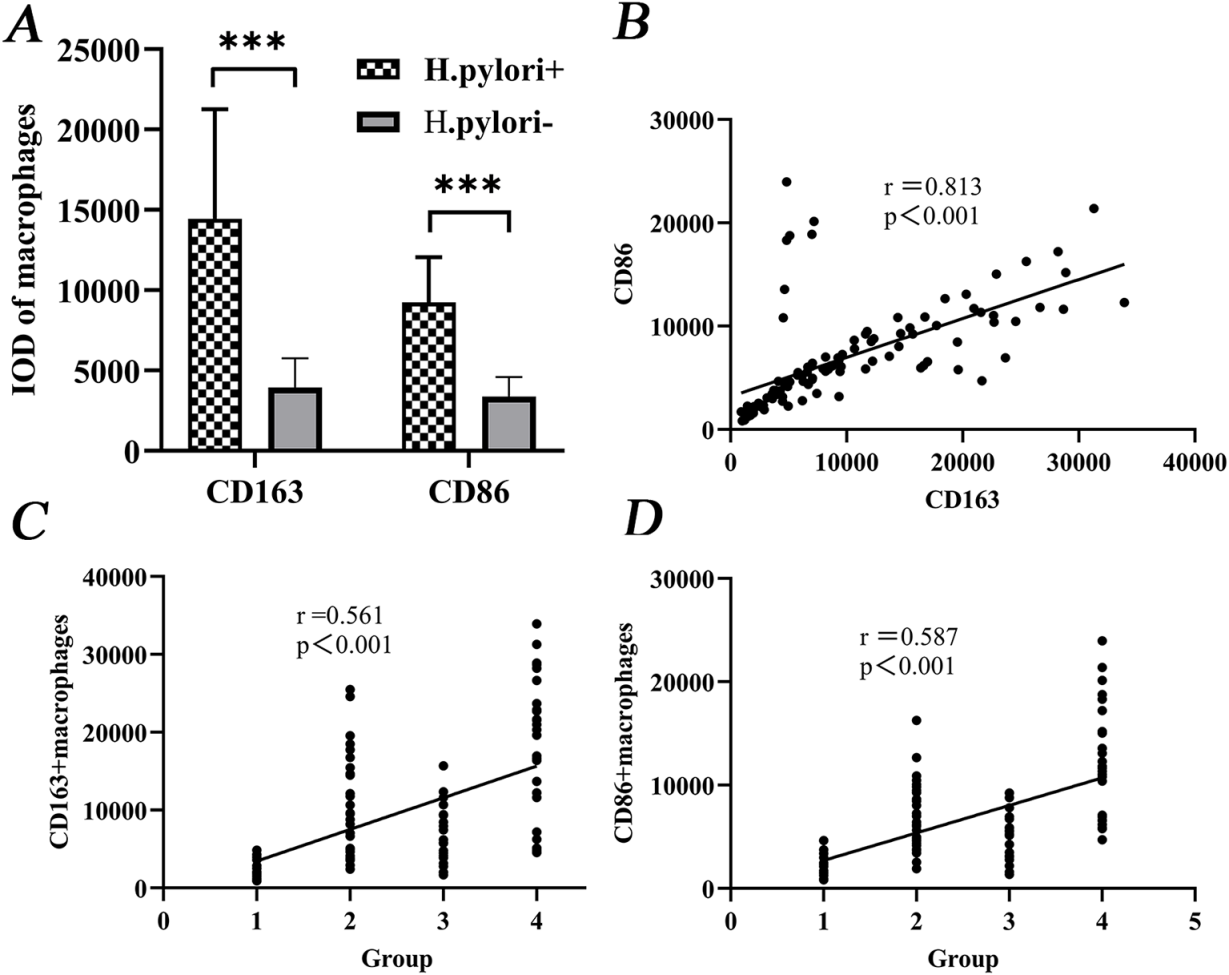
Correlation of CD163^+^/CD86^+^ TAMs levels with CRC development. **(A)** Differential expression between *H.pylori*-infected and uninfected groups. **(B)** Correlation between CD163^+^ and CD86^+^ expression. **(C, D)** Positive correlation of both markers with malignancy grade. Groups: 1, Normal; 2, CAS; 3, SSA; 4, CRC. ***P < 0.001 (MWU test).

CD163^+^ and CD86^+^ TAMs were significantly more abundant in *H.pylori*-positive patients compared with those without infection (*P <* 0.001; **Figure 2A**). A strong positive correlation was found between CD163^+^ and CD86^+^ expression (Spearman’s ρ = 0.813, *P <* 0.001; **Figure 2B**). Both markers were also positively correlated with CRC progression (CD163^+^: ρ = 0.561, *P <* 0.001; CD86^+^: ρ = 0.587, *P <* 0.001; **Figures 2C, D**).

### 3.3 Expression in CRA patients by clinical characteristics

In the CRA group, patients with adenomas ≥1 cm in diameter had higher expression of CD163^+^ and CD86^+^ TAMs than those with smaller adenomas (*P <* 0.05). Similarly, patients with multiple adenomas showed higher expression than those with single adenomas (*P <* 0.05). Patients who were *H.pylori*-positive also had higher marker expression than non-infected individuals (*P <* 0.05) (**Table 2**).

**Table 2:** Expression of CD163^+^/CD86^+^ TAMs and *H. pylori* infection across CRA patient subgroups.

Variable	N (%)	CD163,M (Q_1_,Q_3_)	Statistic	P	CD86,M (Q_1_,Q_3_)	Statistic	P
Total	61 (100)	7065.96 (4483.53,10672.70)			5601.69 (3684.24,8045.12)		
Sex			Z=-0.131	0.896		Z=0.000	1.000
Male	34 (55.74)	7702.38 (4773.17,9427.82)			5580.89 (3780.79,7007.27)		
Female	27 (44.26)	7015.93 (4477.27,13379.70)			5632.93 (3331.30,8710.58)		
Age			Z=-0.247	0.805		Z=-0.261	0.794
< 60	34 (55.74)	7253.25 (4128.22,12354.00)			5580.89 (3427.60,8784.28)		
≥ 60	27 (44.26)	7055.07 (5791.30,9578.44)			5632.93 (4482.40,7086.22)		
Site			Z=-0.073	0.942		Z=-0.054	0.957
Rectum	12 (19.67)	7442.74 (3873.30,12539.05)			4997.73 (3783.96,7924.32)		
Colon	49 (80.33)	7065.96 (4647.59,10658.54)			5601.69 (3503.18,8464.31)		
Diameter			Z=-6.022	<.001		Z=-6.311	<.001
<1 cm	32 (52.46)	4565.56 (3645.91,6652.60)			3732.52 (2921.31,4746.60)		
≥1 cm	29 (47.54)	11626.15 (8781.84,15454.57)			8464.31 (6173.92,9480.48)		
Number			Z=-6.115	<.001		Z=-5.690	<.001
Solitary	25 (40.98)	4128.22 (3139.36,4926.54)			3503.18 (2746.34,4362.52)		
Multiple	36 (59.02)	9578.44 (7702.38,14577.13)			7046.79 (5738.68,9268.61)		
Obesity			Z=-2.388	0.017		Z=-1.288	.198
yes	28(45.90)	8528.66(7026.26,11142.35)			5990.63(4371.46,8569.32)		
no	33(54.10)	5764.89(3699.20,10672.70)			5138.11(3494.33,7803.52)		
*H.pylori*			Z=-5.416	<.001		Z=-5.705	<.001
Infected	29(47.54)	10672.70 (8194.00,15454.57)			7803.52 (6038.08,9480.48)		
Uninfected	32(52.46)	4565.56 (3645.91,6864.03)			3732.52 (2921.31,4746.60)		

Z: Mann-Whitney test; M: Median; Q_1_: 1st Quartile; Q_3_: 3rd Quartile.

### 3.4 Expression in CRC patients by clinical characteristics

In CRC patients, CD163^+^ TAM expression was significantly higher in stages III–IV compared with I–II (*P <* 0.05), in those with lymph node metastases (*P <* 0.05), and in patients with low tumor differentiation (*P <* 0.05). CD86^+^ TAM expression was lower in patients at stages III–IV and in those with metastases or low differentiation (all *P <* 0.05). Both markers were expressed at higher levels in *H.pylori*-infected patients (*P <* 0.05) (**Table 3**).

**Table 3:** Expression of CD163^+^/CD86^+^ TAMs and *H. pylori* infection in CRC patient subgroups.

Variable	n (%)	CD163,M (Q_1_,Q_3_)	Statistic	P	CD86,M (Q_1_,Q_3_)	Statistic	P
Total	29 (100)	19614.60 (7182.47,22899.10)			11644.53 (6618.20,15188.14)		
Sex			Z=-1.594	0.117		Z=-1.373	0.180
Male	12 (41.38)	14434.26 (4841.20,22105.68)			12642.80 (10927.33,16746.85)		
Female	17 (58.62)	20303.61 (13682.83,26651.32)			10396.24 (5969.45,13086.00)		
Age group			Z=-0.330	0.764		Z=-0.236	0.835
< 60	9 (31.03)	16639.37 (13682.83,21559.32)			11822.28 (7094.63,13572.10)		
≥ 60	20 (68.97)	20291.80 (6723.18,23286.39)			11337.34 (6584.85,16746.85)		
Site			Z=-0.094	0.945		Z=-0.754	0.472
Rectum	9 (31.03)	16390.73 (7182.47,28215.57)			11644.53 (5969.45,13572.10)		
Colon	20 (68.97)	19959.11 (9320.94,22804.17)			11528.45 (7019.85,16746.85)		
TNM stage			Z=-2.575	0.009		Z=-2.095	0.037
I-II	14 (48.28)	7100.39 (4841.94,21559.32)			15107.70 (10824.51,18771.47)		
III-IV	15 (51.72)	21645.88 (18287.86,25162.50)			10396.24 (6260.48,11767.89)		
LN metastasis			Z=-2.182	0.029		Z=-2.182	0.029
Negative	15 (51.72)	7182.47 (4971.80,22229.21)			15027.25 (11083.95,18538.51)		
Positive	14 (48.28)	21307.44(16961.12,23673.68)			8745.44 (5969.45,11713.50)		
Histology			Z=-3.822	<0.001		Z=-2.956	0.002
Low	22 (75.86)	21602.60 (16639.37,26551.32)			10713.19 (6181.94,12294.42)		
Middle -High	7 (24.14)	4841.94 (4749.17,6059.98)			18771.47 (15938.83,19515.05)		
Obesity			Z=-4.099	<0.001		Z=-2.147	0.032
yes	21(72.41)	21645.88(16961.12,26651.32)			11030.14(6551.50,12294.42)		
no	8(27.59)	4971.80(4749.17,6641.10)			18538.51(12198.31,195515.05)		
*H.pylori*			Z=-2.077	0.037		Z=-2.005	0.045
Infected	26(89.66)	20636.31 (12229.14,23673.68)			11767.89 (6945.07,17212.47)		
Uninfected	3(10.34)	6263.88 (5402.55,8943.73)			5869.24 (5261.12,8346.88)		

Z: Mann-Whitney test; M: Median; Q_1_: 1st Quartile; Q_3_: 3rd Quartile.

### 3.5 mIF analysis confirmed significant enrichment of TAMs in CRC tissues

**Figure 3:**
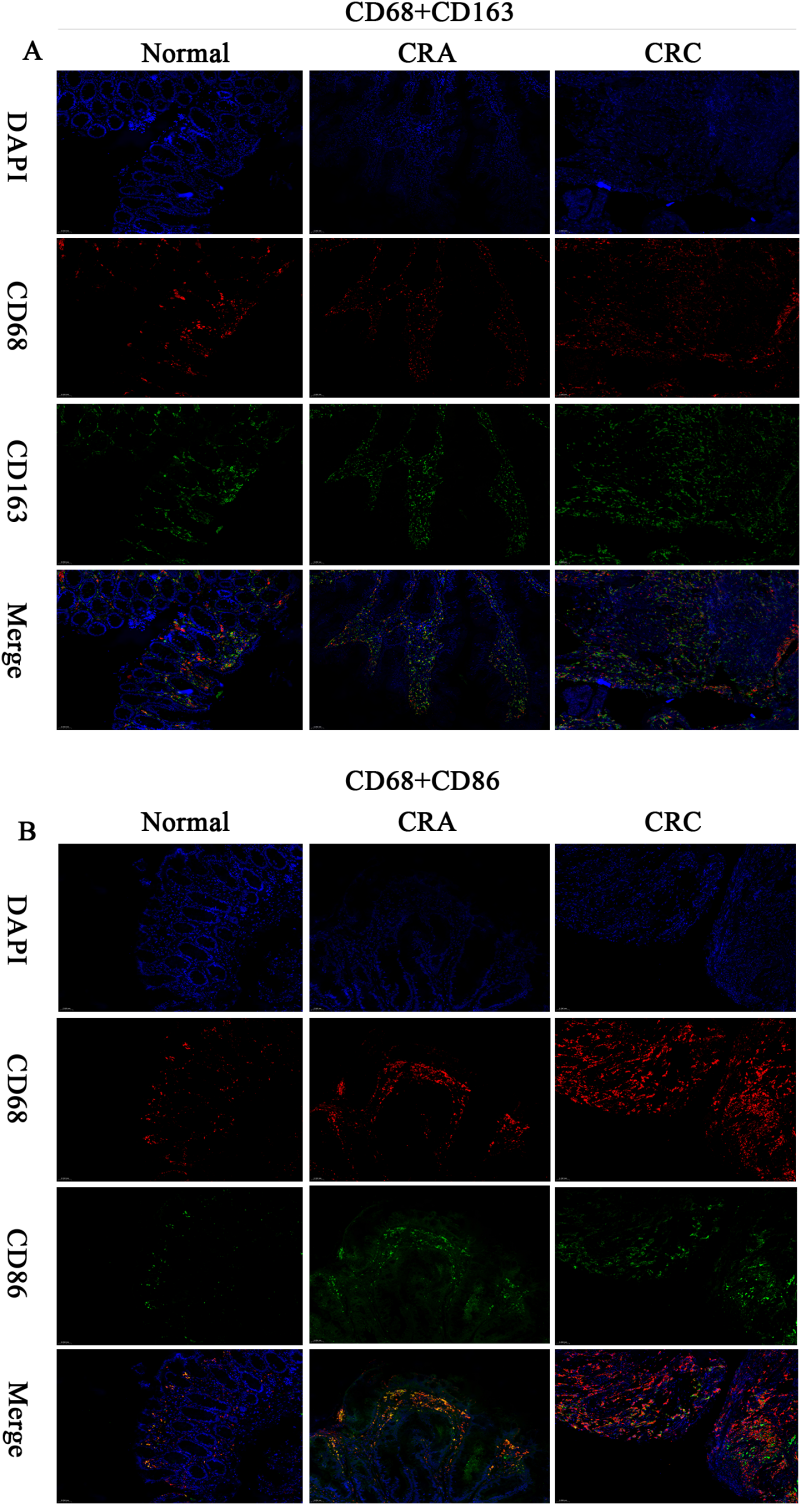
Multiplex immunofluorescence (200×) : CD68^+^ (red), CD163^+^ (green), CD86^+^ (green), DAPI (nuclei, blue), Merge (multichannel overlay), Scale bar: 50 μm. **(A)** CD68^+^CD163^+^ dual-positive cells (IF). **(B)** CD68^+^CD86^+^ dual-positive cells (IF).

**Figure 4:**
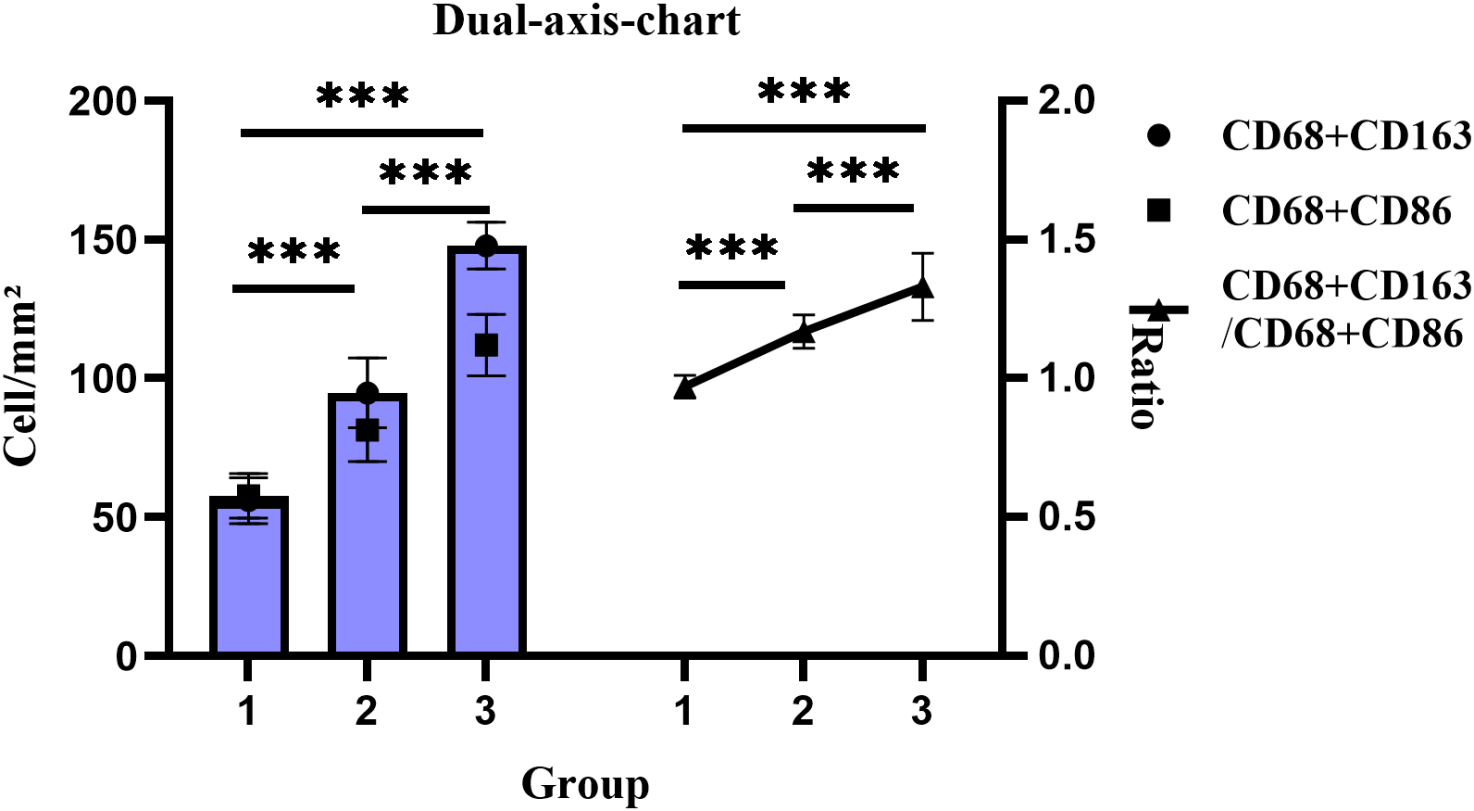
Changes in cell density and proportion of CD68^+^CD163^+/^CD68^+^CD86^+^ dual-positive TAMs across groups. Groups: 1, Normal; 2, CRA; 3, CRC. ***P < 0.001 (Tukey HSD).

mIF-based quantitative analysis revealed significantly higher counts of CD68^+^CD163^+^ and CD68^+^CD86^+^ TAMs and an elevated CD68^+^CD163^+/^CD68^+^CD86^+^ TAMs ratio in the CRC cohort (n=6) compared to the CAS cohort (n=10) and normal controls(n=4). The overall trend in tissue expression was as follows: CRC > CRA > Normal (all P < 0.001). (**Figures 3**, **4**).

### 3.6 Multiple linear regression in CRA and CRC groups

Multiple linear regression was performed using CD163^+^ and CD86^+^ TAMs expression as dependent variables. For the CRA group, independent variables included adenoma size, number, obesity, age, sex, site, and *H.pylori* infection status. For the CRC group, independent variables included age, sex, site, tumor differentiation, lymph node metastasis, TNM stage, obesity, and *H.pylori* infection status. The results indicated that expression levels were significantly associated with adenoma diameter in CRA, and with differentiation, obesity, and *H.pylori* infection in patients with CRC (**Table 4**).

**Table 4:** Multiple linear regression analysis of factors associated with CD163^+^/CD86^+^ TAM expression in CRA and CRC patients.

Group	Variables	β	S.e	T	P	β (95%CI)
CRA-CD86	Diameter					
	< 1cm					Reference
	≥1 cm	4488.870	505.200	8.885	<.001	4488.870 (3477.967 ~ 5499.773)
CRA-CD163	Diameter					
	<1 cm					Reference
	≥1 cm	7545.441	984.454	7.665	<.001	7545.441 (5575.552 ~ 9515.329)
CRC-CD86	*H.pylori*					
	Uninfected					Reference
	Infected	6228.716	2493.190	2.498	0.019	6228.716 (1103.890 ~ 11353.542)
	Histology					
	Low					Reference
	Middle -High	7991.376	1774.363	4.504	<.001	7991.376 (4344.122 ~ 11638.631)
CRC-CD163	Obesity					
	no					Reference
	yes	16405.198	2206.201	7.436	<.001	16405.198 (11878.446 ~ 20931.949)

Stepwise (bidirectional) regression method.

## 4 Discussion

The transition from CRA to CRC is a prolonged process that requires several decades. Therefore, early detection and resection of precancerous lesions are critical for preventing progression of CRC. TAMs have been implicated in the pathogenesis of *H.pylori*-related gastric malignancies (18). Wei et al. demonstrated that *H.pylori* can activate the JAK1/STAT1 signaling pathway in macrophages, leading to CCL3 secretion and subsequent gastric mucosal damage (19). Furthermore, *H.pylori* promotes gastric carcinogenesis by enhancing interleukin (IL)-6-mediated autocrine and paracrine loops between macrophages and gastric epithelial cells (20). Recent studies have also linked *H.pylori* infection to an elevated risk of CRC (21–24). Notably, CRC incidence decreases significantly following *H.pylori* eradication (25). Ralser et al. found that Helicobacter pylori infection accelerates tumorigenesis, concomitant with depletion of regulatory T (Treg) cells and pro-inflammatory T cells, goblet cell deficiency, and STAT3 pathway activation (26). Luo et al. further reported that *H.pylori*-induced temperate phage expansion and microbiota interactions may contribute to CRC pathogenesis (27). Our study found that *H.pylori* infection rates were highest in CRC patients, followed by those with CRA, and lowest in the normal group, suggesting a positive correlation between infection and disease severity. This supports the hypothesis that *H. pylori* may play a role in CRC development.

CD163 is predominantly expressed on monocytes and tissue macrophages, Prior studies indicate that CD163^+^ TAMs may induce regulatory T cell expansion via IL-10/TGF-sl thereby exhausting effector T cells. Numerous studies have reported that CD163 is increased in different cancers and is associated with poor prognosis due to its tumor-promoting functions (28–30). For example, Lira et al. demonstrated that M2-like macrophages facilitate cervical cancer progression via the STAT3/NF-κB pathway (31), whereas Ito et al. showed that in CRC, M2-like macrophages enhance their resistance to oxidative stress through the Nrf2/HO-1 axis, promoting survival in the TME (32). CD86 is an immunophenotype associated with antitumor immune modulation, Evidence suggests that CD86^+^ TAMs may inhibit CD8^+^ T cell activation through PD-L1 co-expression. Teng et al. reported that CASC19 transferred from M1-like macrophages to colon cancer cells via exosomes inhibited tumor cell proliferation and migration by targeting miR-410-3p (33). A reduction in M1-like macrophages infiltration has also been associated with poor prognosis in right-sided CRC patients (34). This study revealed progressively elevated CD163^+^ and CD86^+^ TAMs levels in colorectal tissues: normal mucosa < CRA < CRC, suggesting their potential involvement in immune modulation during tumor progression. These findings offer insights for developing potential immunotherapeutic and prognostic strategies in CRC.

Several studies have suggested a link between *H.pylori* infection and macrophage polarization. Peng et al. found that *H.pylori* induced indoleamine 2,3-dioxygenase expression and M2-like polarization (14). Lu et al. reported that in all stages of gastric lesions, patients who are *H.pylori*-positive showed higher expression of CD86 and CD206 compared with *H. pylori*-negative individuals (13). our study found elevated levels of CD163^+^ and CD86^+^ TAMs in *H.pylori*-infected patients. This suggests that *H.pylori* may recruit TAMs through specific pathways, with testable hypotheses including: (i) *H.pylori* drives Treg-mediated immunosuppression by recruiting CD163^+^ TAMs, verifiable via mIF assessing spatial co-localization between CCL19^+^ epithelial cells and CD163^+^ TAMs; (ii) CD86^+^ TAMs in CRC patients lose antigen-presenting capacity and undergo transformation into a ‘pseudo-activated’ state, testable through flow-sorted CD86^+^ TAMs co-cultured with CD8^+^ T cells followed by PD-1 expression analysis. Thus, elevated CD163^+^CD86^+^ TAM levels may associate with T-cell dysfunction, though their causality and *H.pylori’s* direct role require further validation.

Zhang et al. found that CD163^+^ macrophages promote polyp progression in pediatric patients by inhibiting T-cell responses via TGF-β production (35). Peyravian et al. showed that CD86 expression positively correlated with dysplasia in polyps (36). Our univariate analysis indicated that patients with adenomas ≥1 cm or multiple adenomas exhibited significantly higher expression of both CD163^+^ and CD86^+^ TAMs. Multivariate regression further confirmed this relationship: CD163^+^ TAMs (β = 7545.441, 95% CI: 5575.552 ~ 9515.329) and CD86^+^ TAMs (β = 4488.870, 95% CI: 3477.967 ~ 5499.773) were significantly increased in patients with larger adenomas. Our univariate analysis found that in CRC patients, lower differentiation, advanced TNM stage, and lymph node metastasis were associated with higher expression of CD163^+^ TAM and lower expression of CD86^+^ TAM. Consistent with previous studies (37, 38). These results suggest that TAMs expression reflect disease severity and may contribute to assess malignancy risk. The M2-like/M1-like macrophages ratio is a useful prognostic indicator, Fadhil et al. reported higher M2-like/M1-like macrophages ratios in breast cancer patients than in controls (39), and Parekh et al. found similar results in advanced oral submucous fibrosis and squamous cell carcinoma (40). Our study further demonstrated a progressive increase in the CD68^+^CD163^+^/CD68^+^CD86^+^ ratio from normal tissue through CRA to CRC, highlighting the role of macrophage phenotypic switching and imbalance in TME homeostasis during carcinogenesis. Additionally, the body mass index is a known risk factor for CRC incidence and mortality (41). Obesity may enhance macrophage activation by suppressing miR-192 expression (42). In CRC groups, patients with obesity exhibited higher CD163^+^ TAM expression. Multivariate regression confirmed this association in CRC (β = 16405.198, 95% CI: 11878.446 ~ 20931.949), suggesting that obesity-induced TME changes may contribute to CD163^+^ TAM enrichment and tumorigenesis.

This study also has several limitations:(i) The single-center, cross-sectional design precludes determination of the temporal sequence and causal relationships between *H.pylori* infection and TAMs;(ii) The relatively small sample size in the CRC cohort may compromise the precision and statistical power of the multivariate regression model. Although stepwise regression was employed to mitigate overfitting, the findings should be interpreted with caution and validated in larger cohorts;(iii) Elevated *H.pylori* prevalence (~90%) in the CRC cohort risks overestimating its effects on TAMs polarization, compromising findings’ generalizability;(iv) Reliance on UBT as the sole detection method may introduce non-differential misclassification, potentially attenuating effect sizes. Due to sample size limitations, we could not quantify bias severity by excluding high-risk misclassified subsets. Future studies should incorporate *H.pylori* stool antigen testing to comprehensively enhance diagnostic accuracy; (v) the coverage of macrophage marker panels was limited; (vi) residual confounding factors were not fully controlled, in addition, multiple comparisons introduced statistical inference risks.

Future studies should: (i) Validate and refine the TAMs differentiation model in expanded prospective cohorts stratified by *H.pylori* infection status; (ii) Establish a multimodal *H.pylori* detection framework integrating stool antigen testing, PCR genotyping, and culture verification; (iii) Spatially resolve TAM-tumor cell crosstalk networks under *H.pylori*-positive conditions using transcriptomics; (iv) Verify *H.pylori*-mediated TAMs regulation via flow-sorted subpopulations and organoid co-cultures targeting candidate pathways identified in (3).

In conclusion, this study found that CD163^+^ and CD86^+^ TAMs are significantly increased in CRC and are associated with *H. pylori* infection. Their expression correlates with lesion severity, indicating a potential role in CRC pathogenesis. However, further studies are warranted to elucidate their exact mechanisms in colorectal tumor development and progression.

## Data Availability

The raw data supporting the conclusions of this article will be made available by the authors, without undue reservation.
